# Carbon and Nitrogen Stable Isotope Variation in Semi‐Arid Woody Plant Species Growing in Managed and Natural Settings Across Southern California

**DOI:** 10.1002/pei3.70149

**Published:** 2026-04-22

**Authors:** R. Brandon Pratt, George L. Vourlitis, Monica Uriaz, Marlem Lopez‐Meza, Adam Sanders, Ethan Esparza, Ava Santos‐Volpe, Calvin Kamimura, Cinzia Fissore, Ehren Moler, Dustin VanOverbeke, Wallace M. Meyer

**Affiliations:** ^1^ California State University Bakersfield Department of Biological Sciences Bakersfield California USA; ^2^ California State University San Marcos Department of Biological Sciences San Marcos California USA; ^3^ California Polytechnic State University at Pomona Department of Biological Sciences Pomona California USA; ^4^ University of Redlands Department of Biology Redlands California USA; ^5^ Pomona College Biology Department Claremont California USA; ^6^ Whittier College Department of Biology and Environmental Science Program Whittier California USA

**Keywords:** δ^13^C and δ^15^N natural abundance, urban landscapes, water use efficiency

## Abstract

Landscape planners in semi‐arid and arid regions often plant native shrubs to reduce water and fertilizer use; however, landscape management practices might alter shrub resource use and make them more dependent on irrigation and fertilization. Our objective was to assess whether water and nutrient subsidies in managed landscapes affected plant resource use. We measured carbon (δ^13^C) and nitrogen (δ^15^N) stable isotopes and tissue N concentrations in leaves of native Mediterranean‐type vegetation growing in adjacent urban and natural areas at six different universities throughout Southern California. These data are used to test the hypothesis that native shrubs growing in natural areas with no or infrequent irrigation would have higher δ^13^C but lower δ^15^N values than conspecifics growing in managed landscapes. Leaf δ^13^C declined as aridity increased across our six campuses, but only for plants growing in natural areas, suggesting that irrigation decoupled water use efficiency (WUE) from natural climate conditions. Plants growing in urban landscapes had lower δ^13^C values than conspecifics growing in natural areas, suggesting that irrigated plants had lower WUE. Plants also had higher leaf δ^15^N in managed settings, suggesting that fertilization and irrigation increased N availability and mobility. Our results indicate that semi‐arid plants that normally exhibit stomatal control over water loss in natural areas might be more sensitive to drought if irrigation systems fail or are shut off due to high water costs. Using native plants in urban landscapes may not result in water savings because native plants are less efficient in their water use.

## 1 Introduction

Plants growing in urban landscapes are often exposed to novel soils (Pouyat et al. 2010; Vourlitis et al. 2022), communities (Pickett et al. 2011; Aronson et al. 2014), climates (Wilson 2020; Lan et al. 2022), and resource subsidies (Wang et al. 2017; Landon et al. 2025), which can affect their growth and resource use. For example, plants growing in urban environments are commonly exposed to the heat island effect, which causes declines in photosynthesis and leads to photoinhibition when temperatures exceed the physiological optimum (Percival 2023). Warmer temperatures also increase leaf‐atmosphere vapor pressure deficit and reduce stomatal conductance (Percival 2023). However, plants growing in urban landscapes are often irrigated, which can reduce the risk of direct heat‐related impacts on photosynthesis and stomatal conductance, especially in semi‐arid and arid environments (Machado and Paulsen 2001). When plants have access to water, stomatal conductance may not be as sensitive to heat as plants are able to evaporatively cool leaves (Kolb and Robberecht 1996). However, high vapor pressure deficits that develop during heat waves will eventually cause stomatal closure because high transpiration rates can cause extreme negative xylem tensions and increase the danger of xylem embolisms (Sperry et al. 2016).

Understanding plant water use in irrigated landscapes can improve water resource management, especially in arid and semi‐arid environments where irrigation may become unsustainable as water scarcity and costs increase (Tabassum et al. 2021). One approach to reduce irrigation is to use more native, drought‐resistant plants in urban landscapes (Zollinger et al. 2006; Tabassum et al. 2021). Some plant species avoid drought by having deep root systems that can access more stable belowground water resources or by closing stomata or shedding leaves to reduce transpiration. Plants that close their stomata might have a higher water use efficiency (WUE), meaning that they have higher rates of growth (or photosynthesis) per unit water lost (Pronger et al. 2019). Other plants, classified as anisohydric, might tolerate drought by continuing to transpire as soils dry and they experience tissue dehydration (Poole and Miller 1975; Martínez‐Vilalta et al. 2014), thus, these species would be expected to have lower WUE. In addition to water use strategies of differing plants, WUE is also affected by environmental conditions; for example, drought adapted plants growing in irrigated urban environments may have different WUE than conspecifics growing in natural environments (Hartman and Danin 2010), which would reduce their ability to conserve water.

Plants growing in urban landscapes are also commonly subject to higher concentrations of pollutants that can affect their physiology. For example, some pollutants (e.g., reactive NO_x_) can increase in urban settings due to fossil fuel burning and fertilizer use (Fenn et al. 2003), and since plant growth is often N limited (Vitousek and Howarth 1991), atmospheric inputs of N may stimulate rates of plant growth (Chen et al. 2015; Vourlitis et al. 2021) provided that N input does not exceed biological demand (Fenn et al. 2010). Furthermore, direct fertilization of urban vegetation may also partially alleviate negative impacts from heat stress (Fu and Huang 2003; but see Percival 2023). Regardless, vegetation in urban landscapes exposed to nutrient subsidies is likely to alter N uptake and photosynthesis in areas where native soils are nutrient poor.

Environmental variation in plant water and nutrient use can be traced in part using stable isotopes (Flanagan et al. 1992; Dawson et al. 2002). For example, there is a positive correlation between leaf δ^13^C and WUE (Flanagan et al. 1992; Dawson et al. 2002) because there is a reduction in the ratio of internal to external CO_2_ concentration as stomatal conductance declines, which causes a decline in C isotope discrimination (^13^Δ) and a higher δ^13^C (Farquhar et al. 1982). Thus, plants growing under low water availability would have lower stomatal conductance, higher (less negative) δ^13^C, and higher WUE.

High foliar δ^15^N values have been found to be positively correlated with atmospheric N deposition, rates of N mineralization, leaching, and other processes that are enhanced by added N (Högberg 1997; Korontzi et al. 2000; Dawson et al. 2002; Pardo et al. 2013; Chou et al. 2026). If N cycling and uptake are enhanced by N inputs in urban landscapes, leaves of native species growing in managed urban landscapes should have a higher δ^15^N than their conspecifics growing in natural areas. This has been shown in many studies where long‐term exogenous N inputs have increased rates of N cycle processes and caused ^15^N enrichment of soil, litter, and plant tissue (Högberg 1997; Korontzi et al. 2000; Chou et al. 2026).

Here we report the leaf δ^13^C and δ^15^N natural abundances and tissue N concentrations of native Mediterranean‐type vegetation growing in adjacent urban and natural areas at six different college campuses distributed widely throughout Southern California that span a large gradient in aridity. Leaves from several native shrubs growing in both managed and natural areas were collected in the spring of 2024 to test the hypothesis that native shrubs growing in areas with no, or substantially less, supplemental irrigation or fertilization (referred to as “natural areas”) would have higher δ^13^C but lower δ^15^N natural abundances than conspecifics growing in managed urban landscapes that are subsidized by irrigation and fertilization (referred to as “managed areas”).

## 2 Materials and Methods

### 2.1 Site Descriptions and Sample Collection

The study was performed between January and May 2024 at six different universities: California State University, Bakersfield (CSB), University of Redlands (UR), California State Polytechnic University, Pomona (CPP), Pomona College (PC), Whittier College (WC), and California State University San Marcos (CSM) (see Table 1 for meta‐data). Five of these areas have a semi‐arid Mediterranean‐type climate, while CSB has an arid climate (Table 1); however, the study period was exceptionally wet, with all the sites recording precipitation totals that either met or exceeded the long‐term annual totals for each site.

**TABLE 1:** Metadata for the universities collaborating on the research described here.

University (abbreviation)	Coordinates (N:W)	Elevation (m)	Min–Max temperature (°C)	Precipitation (mm)	ET (mm)	Aridity index	Soil order	Species sampled
CSU‐Bakersfield (CSB)	35.35:119.11	122	5.3–30.6	182	1470	0.12	Entisols	*Ha*, *Qa, Sn*
CSU‐San Marcos (CSM)	33.13:117.16	227	6.8–29.4	369	1311	0.28	Inceptisols	*Ha*, *Sm, Bp*
Cal Poly Pomona (CPP)	34.06:117.82	414	4.4–27.6	418	1388	0.30	Mollisols	*Ac*, *Ha*, *Qa*, *Sa*, *Sm*
Pomona College (PC)	34.10:117.72	362	4.4–27.6	418	1388	0.30	Entisols	*Ac*, *Ha*, *Qa*, *Sa*, *Sm*
University of Redlands (UR)	34.06:117.16	479	3.2–26.7	310	1490	0.21	Entisols	*Ha*, *Qa*, *Sa*, *Sm, Ef*
Whittier College (WC)	33.98:118.03	40	7.5–27.4	341	1369	0.25	Mollisols	*Ha*, *Qa*, *Sa*, *Sm*

*Note:* Data for minimum and maximum temperature were for obtained from the Western Regional Climate Center (www.wrcc.edu) and the U.S. National Weather Service (www.weather.gov) for January–June 2024. Annual precipitation and Hargreaves evapotranspiration (ET) are for the period of 1981–2023 using PRISM 4 km data (https://www.climateengine.org/). The aridity index (AI) is calculated as precipitation/ET. Data for soil order were obtained from the U.C. Davis Soil Web (https://casoilresource.lawr.ucdavis.edu/, accessed June 18, 2024). Species sampled were *Artemisia californica* (*Ac*), *Baccharis pilularis* (*Bp*), *Eriogonum fasciculatum* (*Ef*), *Heteromeles arbutifolia* (*Ha*), *Quercus agrifolia* (*Qa*), *Salvia apiana* (*Sa*), and *Salvia mellifera* (*Sm*), and *Sambucus nigra* (*Sn*).

At Pomona College (PC), leaf samples of *Salvia mellifera, S. apiana*, *Heteromeles arbutifolia* , *Artemisia californica* , and *Quercus agrifolia* (*n* = 6–8 individuals for each species/habitat) were collected from managed landscapes on the main campus and from natural areas at the Robert J. Bernard Field Station located about 1.5 km north of the main campus. CSU Bakersfield (CSB), leaf samples of *H. arbutifolia* , *Q. agrifolia* , and *Sambucus nigra ssp. caerulea* (*n* = 8 individuals for each species/habitat) were collected from managed areas on the main campus and from natural areas within ca. 22.5 km of CSB. Because of the arid climate, natural areas near CSB received limited drip irrigation; however, managed populations received abundant irrigation and were associated with lawns and other water‐loving landscape plants. At the University of Redlands (UR), leaf samples of *Q. agrifolia* , *H. arbutifolia* , *S. apiana* , and *S. mellifera* (*n* = 8 individuals for each species/habitat) were collected from managed landscapes on the main campus and the Sustainable University of Redlands Farm (SURF), which is a community farm on campus, while leaf samples from natural areas were collected from native vegetation close to the main campus. At California Polytechnic University Pomona (CPP), leaf samples from *Q. agrifolia* , *H. arbutifolia* , *S. apiana* , *S. mellifera* , and *A. californica* (*n* = 8 individuals for each species/habitat except *S. apiana* *n* = 6) were collected in managed and natural (coastal sage scrub) areas located on the main campus. At Whittier College (WC), leaf samples from *S. mellifera, S. apiana, H. arbutifolia*, and *Q. agrifolia* (*n* = 6–8 individuals for each species/habitat for *H. arbutifolia*, and *Q. agrifolia* and 3–8 for *S. mellifera* and *S. apiana* ) were collected in managed areas on the main campus and in natural areas along the Arroyo Pescadero hiking trail (55 m asl). Lastly, at CSU San Marcos (CSM) leaf samples of *Baccharis pilularis* , *S. mellifera* and *H. arbutifolia* (*n* = 8 individuals for each species/habitat) were collected from managed landscapes and in natural areas (mixed chaparral) located on the main campus.

Approximately 5–10 leaves were collected from each individual sampled, and leaves were collected at least three nodes deep on branches to avoid collecting newly grown leaves. Adult leaf tissue was collected from the outer edge of the southern face of the plant where the plant received more sunlight.

### 2.2 Climate Data

Data for minimum and maximum temperature were obtained from the Western Regional Climate Center (www.wrcc.edu) and the U.S. National Weather Service (www.weather.gov) for January–June 2024. Long‐term data for precipitation and Hargreaves Evapotranspiration were compiled from the climate engine website (https://www.climateengine.org/) and using PRISM 4 km data (Table 1). Annual precipitation and Hargreaves Evapotranspiration were taken as annual means between 1981 and 2023 that were averaged over a 12‐month water year from July to June. The aridity index of each site was calculated as precipitation divided by evapotranspiration (Table 1).

### 2.3 Laboratory Analyses

Leaf samples were oven‐dried at 40°C for 72 h, and dried leaf samples were ground using a Wiley Mill, Ball Mill, or mortar and pestle depending on the lab where samples were processed. Approximately 2.5–3.5 mg of the ground sample was weighed into an aluminum capsule using forceps, and the samples were assessed for total C and N using an elemental analyzer (Elementar vario MICRO cube, Mt. Laurel, New Jersey) and δ^13^C and δ^15^N isotopic ratios using a continuous‐flow isotope‐ratio mass spectrometer (Delta V Plus, Thermo Scientific, Bremen, Germany).

### 2.4 Statistical Analysis

Data were analyzed as linear mixed models using the R package glmmTMB (Brooks et al. 2017), or in the case where there were no random treatment factors as ANOVAs. Response variables included δ^13^C, δ^15^N, and leaf N % with the focal treatment factor of natural and managed habitats. The treatment factor was analyzed across additional levels of treatments including sites (6 locations) and different species (9 total). Species were treated as random factors nested within site. Model assumptions were tested using R package DHARMa (Hartig 2022). Type II Wald χ^2^ tests were used to assess significance of predictors for mixed models and marginal means, generated with the emmeans package, were used for descriptive statistics and Tukey post hoc tests (Lenth 2024). Some species were sampled across multiple sites, and this allowed us to test for a habitat effect while holding species constant, which were run as separate linear models. Assumptions of normality and constant variance were tested, and data were log‐transformed, or generalized linear models with a log‐normal distribution were used as necessary to meet assumptions. Type III SS were used when significant interactions were observed; otherwise, type II SS were used.

## 3 Results

### 3.1 Variation in Treatment Effects Across Universities

**FIGURE 1:**
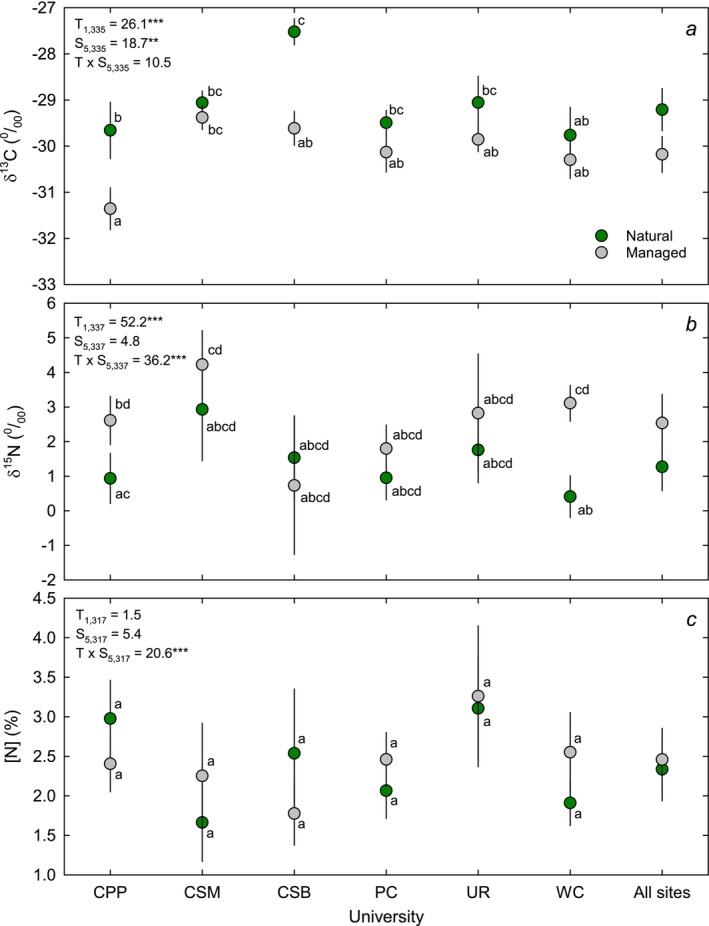
Mean (±SE) leaf δ^13^C (a) and δ^15^N (b) natural abundance and leaf N concentration (c) for native species in natural (green symbols) and managed landscape (gray symbols) habitats on the campuses of Cal Poly Pomona (CPP), CSU San Marcos (CSM), CSU Bakersfield (CSB), Pomona College (PC), University of Redlands (UR), Whittier College (WC), and across all sites. Results from an analysis of deviance (Wald *X* ^2^ statistic and factor and effort degrees of freedom) are shown for treatment (T: Managed vs. natural) and site (S: University) effects and the treatment versus site (T × S) interaction. *p < 0.05; **p < 0.01; ***p < 0.001. Sites with unique letters are significantly different.

**FIGURE 2:**
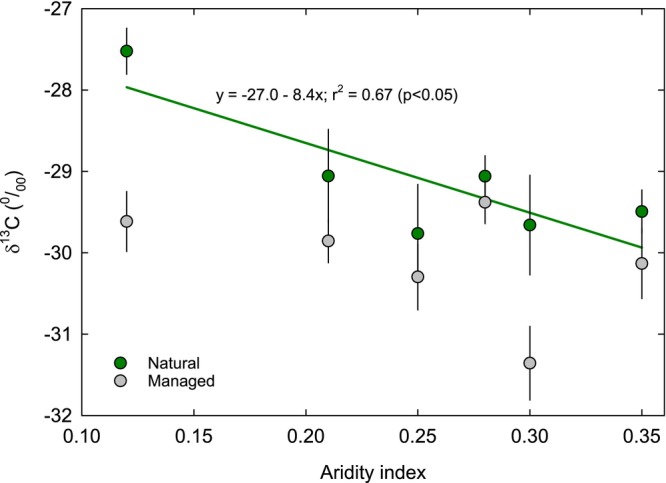
Mean (±SE) leaf δ^13^C (‰) for plants growing in managed (gray symbols) and natural (green symbols) habitats as a function of the aridity index calculated for six universities including Cal Poly Pomona (CPP), CSU San Marcos (CSM), CSU Bakersfield (CSB), Pomona College (PC), University of Redlands (UR), and Whittier College (WC). The aridity index was calculated as annual precipitation/annual evapotranspiration. Regression equation is shown for statistically significant linear trends only (natural systems).

The natural versus managed treatment had a significant effect on δ^13^C (Figure 1a). The marginal means were −29.1‰ for natural and −30.1‰ for managed (Figure 1a), suggesting lower internal CO_2_ relative to ambient during carbon fixation and greater water use efficiency (WUE). Location was also a significant treatment factor. The site differences were associated with aridity (Figure 2), and the site aridity index was negatively correlated with δ^13^C for the natural populations, suggesting high aridity was associated with higher WUE. For the managed populations, the relationship between δ^13^C and aridity index was not significant, suggesting that management practices uncoupled δ^13^C (and WUE) from site aridity (Figure 2).

**FIGURE 3:**
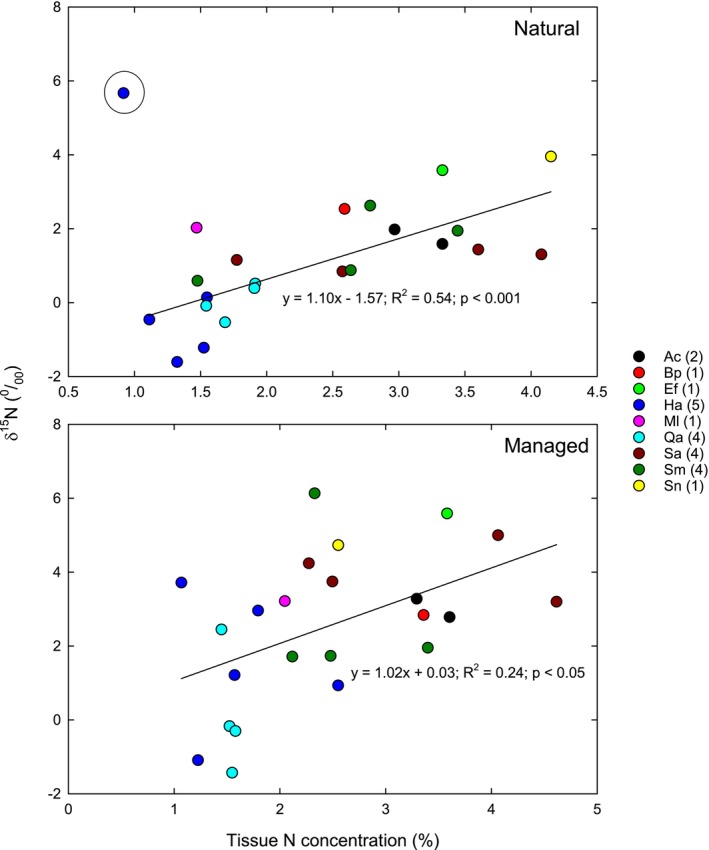
Relationships between leaf δ^15^N natural abundance and leaf tissue concentration for nine species growing in natural (top‐panel) and managed (bottom‐panel) areas on different college campuses across southern California. The number of campuses for each species is indicated in parentheses in the figure legend. The regression for natural areas omits the outlier in the circle. *Artemisia californica* (Ac), *Baccharis pilularis* (Bp), *Eriogonum fasciculatum* (Ef), *Heteromeles arbutifolia* (Ha), *Quercus agrifolia* (Qa), *Salvia apiana* (Sa), *Salvia mellifera* (Sm), and *Sambucus nigra* (Sn).

Leaf δ^15^N was generally higher in managed than natural areas, and there was a significant interaction between treatment and site because only two sites were significantly different (Figure 1b). Site was not a significant predictor for δ^15^N (Figure 1b). In contrast, treatment and site were not important predictors for leaf N concentration; however, there was a statistically significant treatment × species (T × S) interaction (Figure 1c). No site had a significant difference in foliar N between the treatments, and the significant interaction arises because some sites have elevated N in natural areas (CSM, PC, UR, and WC) while other sites show the opposite pattern (CPP and CSB). δ^15^N increased with leaf N in both natural and managed environments, but for natural areas the statistically significant linear relationship was only observed after the *H. arbutifolia* sample from CSM was removed from analysis (Figure 3). This population had the highest values of δ^15^N and the lowest values of tissue N, and we suspect that this sample is an outlier because the natural population was on a steep slope adjacent to a service road where vehicle exhaust would inflate δ^15^N and leaching and runoff would reduce tissue N (Koba et al. 2012; Xu et al. 2019).

**FIGURE 4:**
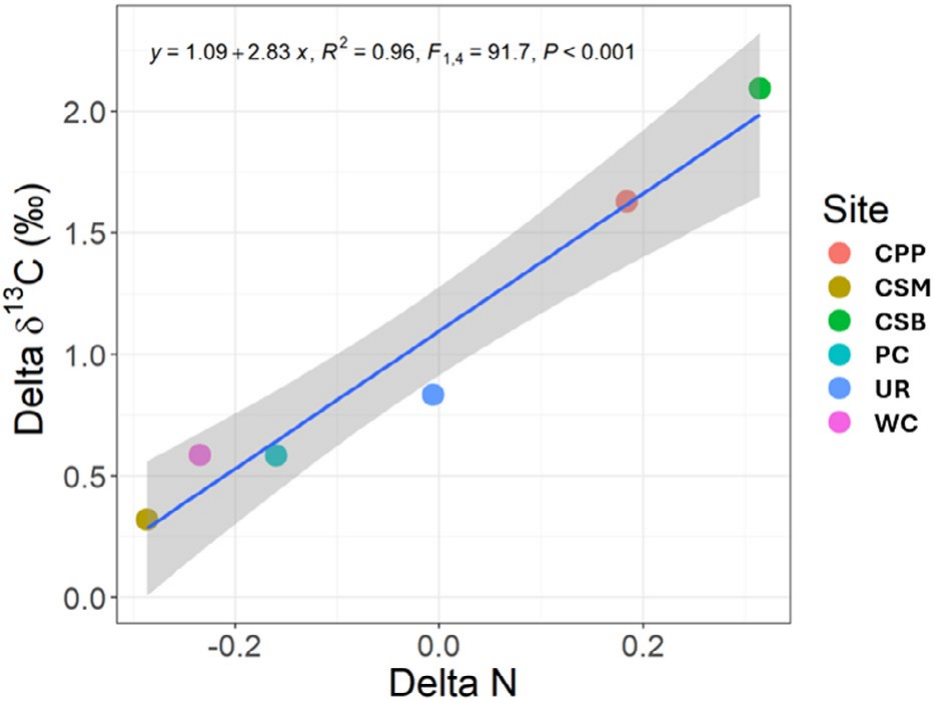
Relationship between the difference (delta) between variables (δ^13^C and N) by treatment (natural–managed) for each site (main effect for site).

The association between δ^13^C and N could help explain site differences in δ^13^C. All sites had larger δ^13^C for the natural treatment, but not all had higher N (Figure 4); therefore, to control for site differences, the difference in δ^13^C and N between natural and managed populations (calculated as natural—managed) for each site was used for the analysis. There was a strong linear relationship where sites with the largest difference in δ^13^C between natural and managed areas also had greater N in natural areas (Figure 4). This suggests a positive link between δ^13^C and N and implicates increased N as a driver of greater δ^13^C.

### 3.2 Variation in Treatment Effects Across Species

**FIGURE 5:**
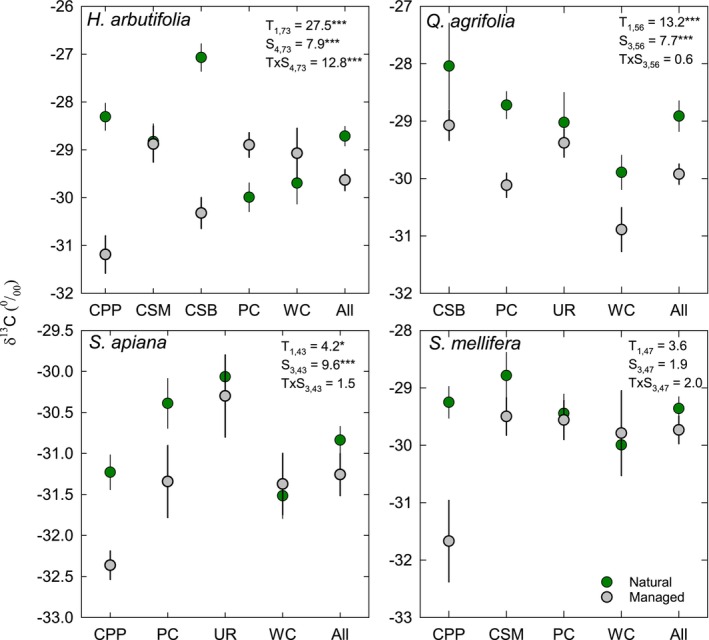
Mean (±SE) leaf δ^13^C natural abundance for *Heteromeles arbutifolia* , *Quercus agrifolia* , *Salvia apiana* , and *Salvia mellifera* growing in natural (green symbols) and managed (gray symbols) areas on 4–6 college campuses depending on species. Results from a 2‐way ANOVA (F‐statistics and factor and error degrees of freedom) are shown for treatment (T: Natural vs., managed) and site (S: University) as the man factors and the T × S interaction. *p < 0.05; **p < 0.01; ***p < 0.001.

Four species were sampled at multiple sites allowing us to compare their responses to managed and natural habitats. For *Heteromeles arbutifolia*, managed habitats had significantly lower δ^13^C (−29.7‰) than natural ones (−28.6‰), but the result was only significant at two sites contributing to an interaction between treatment and site (Figure 5). For *Quercus agrifolia* and *Salvia apiana* , natural populations had higher δ^13^C than managed, but for *Salvia mellifera* there were no significant differences in the δ^13^C (Figure 5).

**FIGURE 6:**
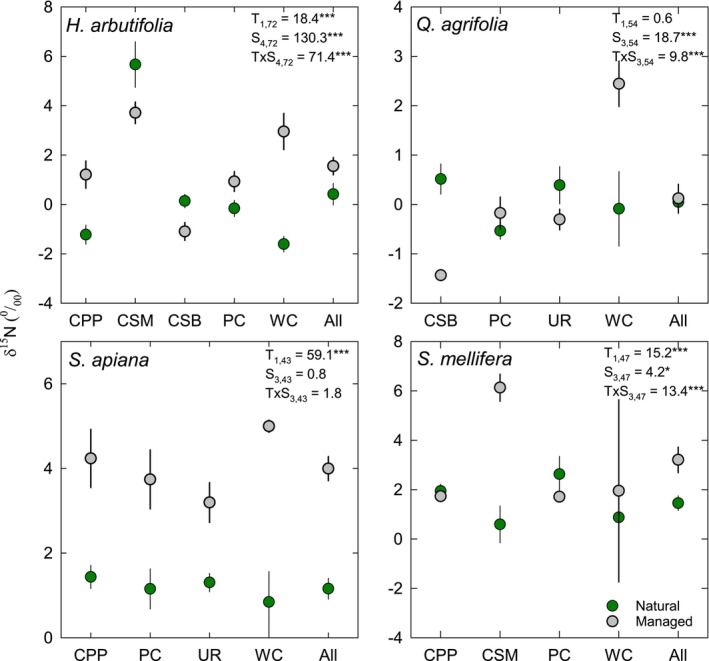
Mean (±SE) leaf δ^15^N natural abundance for *Heteromeles arbutifolia* , *Quercus agrifolia* , *Salvia apiana* , and *Salvia mellifera* growing in natural (green symbols) and managed (gray symbols) areas on 4–6 college campuses depending on species. Results from a 2‐way ANOVA (F‐statistics and factor and error degrees of freedom) are shown for treatment (T: Natural vs. managed) and site (S: University) as the man factors and the T × S interaction. *p < 0.05; **p < 0.01; ***p < 0.001.

The δ^15^N natural abundances of *H. arbutifolia* exhibited a statistically significant T × S interaction because relative differences in δ^15^N were not consistent between managed and natural areas across universities (Figure 6). Plants growing at CSM and CSB had higher δ^15^N values in natural areas while plants on other campuses had the reverse. *Q. agrifolia* also exhibited a statistically significant T × S interaction because plants growing in natural areas had lower or equal values of δ^15^N except at the University of Redlands (Figure 6). *Salvia apiana* exhibited clear differences in δ^15^N, with plants growing in managed areas having consistently higher values of δ^15^N. Variations in δ^15^N natural abundance for *S. mellifera* were significantly higher for managed shrubs at CSM, while relative differences between managed and natural areas were negligible at the other universities, causing a statistically significant T × S interaction (Figure 6).

**FIGURE 7:**
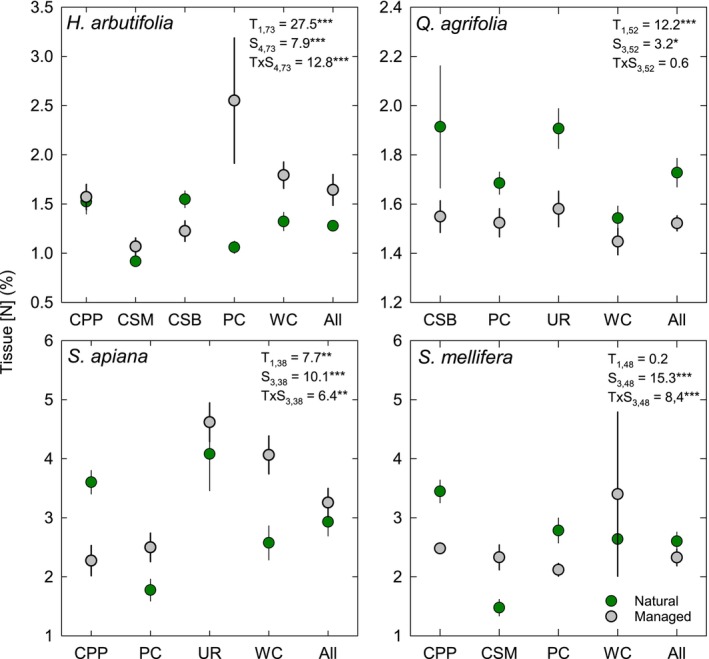
Mean (±SE) leaf tissue N concentration for *Heteromeles arbutifolia* , *Quercus agrifolia* , *Salvia apiana* , and *Salvia mellifera* growing in natural (green symbols) and managed (gray symbols) areas on 4–6 college campuses depending on species. Results from a 2‐way ANOVA (F‐statistics and factor and error degrees of freedom) are shown for treatment (T: Natural vs., managed) and site (S: University) as the man factors and the T × S interaction. *p < 0.05; **p < 0.01; ***p < 0.001.

Variation in tissue N concentration between natural and managed areas was complex, and most species had a statistically significant T × S interaction (Figure 7). For *H. arbutifolia* , plants growing in managed areas had higher tissue N at all sites except CSB. *Quercus agrifolia* was the only species that exhibited a clear treatment effect, with all plants growing in natural areas having higher tissue N than plants growing in managed areas (Figure 7). *Salvia apiana* had higher tissue N in managed areas except for those growing at CPP, while *S. mellifera* had higher tissue N in natural areas except for those growing at CSM and WC (Figure 7).

## 4 Discussion

### 4.1 δ13C Natural Abundances

We hypothesized that leaves of native shrubs growing in natural areas would have higher δ^13^C natural abundances than conspecifics growing in irrigated urban landscapes, which is consistent with our data across universities and for select species. Nevertheless, species‐specific responses were apparent. The plant species studied here have a variety of adaptations to survive drought stress, such as tight control over stomatal conductance and high water use efficiency (WUE) during times of water scarcity, sclerophyllous leaves that resist water loss, deep root systems that stabilize water status, semi‐drought‐deciduous leaves, leaf coverings that reduce solar exposure, or hydraulic architecture that can minimize the potential for xylem cavitation (Gill and Mahall 1986; Galmés et al. 2007; Jacobsen et al. 2007; Pivovaroff et al. 2016). It is likely that increases in WUE for plants growing in natural areas were due to a combination of stomatal control and higher tissue N that presumably was associated with greater rates of net carbon assimilation. This suggests that higher WUE was also affected by biochemical controls on photosynthesis and growth.

We found that only some species had higher δ^13^C in natural areas but differences were not consistent across campuses. This variable response was likely due to the aridity associated with each campus, the frequency in which urban plants were irrigated, and an uncharacteristically wet study period. For example, δ^13^C natural abundances declined along an aridity gradient but only for plants growing in natural areas. This suggests that managed plants exhibited less stomatal control than naturally growing conspecifics. Many of the universities have computerized controls over irrigation that reduce irrigation during times of rainfall; however, even sporadic supplemental irrigation during a very wet spring period was apparently enough to reduce stomatal control and WUE. Presumably, differences in irrigation frequency between universities likely contributed to species‐level deviations from overall patterns. These results indicate that semi‐arid native vegetation exhibits variable WUE depending on natural and/or supplemental water availability.

Stomatal restriction of transpiration can lower intercellular CO_2_ concentrations (C_i_) and increase δ^13^C and WUE. However, another way this can happen is with higher maximum carbon assimilation rates so that when stomata are open, C_i_ is lower, leading to higher rates of ^13^C fixation and greater δ^13^C (Sparks and Ehleringer 1997). We did not measure carbon assimilation rates in our study, but leaf N is positively correlated with maximum rates of carbon assimilation (Field et al. 1983; Wright et al. 2004), which may explain some of the patterns in our data. At the sites where δ^13^C was higher in the natural than managed treatment, the difference in foliar N content was also the highest between natural and managed treatments. These differences in N presumably contributed to the larger differences in δ^13^C between sites and suggest that a combination of stomatal adjustments (cases where N% does not differ between treatments) and increased carbon assimilation capacity (cases where N% is higher in natural areas) contribute to our observed differences in δ^13^C.

### 4.2 δ15N Natural Abundances

We also hypothesized that leaves of native shrubs growing in natural areas would have lower δ^15^N than conspecifics growing in irrigated and fertilized urban landscapes. Our data generally support this hypothesis, as several species had significantly higher δ^15^N values in managed than in natural areas. However, as with δ^13^C, species responses often depended on the university, which presumably reflected the management practices and environmental characteristics unique to each university. Managed areas receive periodic inputs of N‐rich fertilizers, which supply plants with a readily available source of N (Falxa‐Raymond et al. 2014) that is mobilized when urban landscapes are irrigated (Hartman and Danin 2010). While inorganic N fertilizers tend to have a narrow δ^15^N natural abundance that ranges between −2‰ and 2‰ (Bateman and Kelly 2007), leaf δ^15^N values for urban shrubs were often > 2‰, suggesting that other processes were responsible for the ^15^N enrichment of urban plants (Falxa‐Raymond et al. 2014; Ruiz‐Navarro et al. 2016). Nearly all N cycling processes discriminate against ^15^N (Dawson et al. 2002), and if added N speeds up N cycling, which has been reported for many regions exposed to long‐term N addition, faster N cycling in urban areas would also lead to higher ^15^N enrichment in urban plants (Högberg 1997; Korontzi et al. 2000; Ruiz‐Navarro et al. 2016).

Analyzing relationships between δ^15^N natural abundance and leaf N concentration supports the interpretation of faster N cycling in N‐enriched urban landscapes. First, plants growing in managed areas generally had higher foliar δ^15^N than plants growing in natural settings even though the range of tissue N is similar. Secondly, there was a statistically significant positive relationship between tissue N concentration and δ^15^N natural abundance, implying higher ^15^N enrichment in plants with higher N uptake. However, the statistically significant relationship in natural areas was only found after removing an outlier from analysis (*Ha* from CSM), which had lower foliar N concentration but higher δ^15^N values than natural populations of *H. arbutifolia* on other campuses. We feel that both the δ^15^N and tissue N values for this population are outliers because individuals were growing on a steep slope adjacent to a service road. Under these conditions, leaf ^15^N would be enriched from exposure to vehicle exhaust and road dust while N uptake and leaf N concentration would decline due to high rates of N loss from leaching and/or runoff (Koba et al. 2012; Xu et al. 2019).

## 5 Conclusions

Plants growing in urban landscapes had lower δ^13^C values than conspecifics growing in natural areas, and while this pattern depended on the species and university, it was clear that plants growing in natural areas had higher WUE than plants growing in urban areas. These differences were apparent even during an extremely wet year, which presumably reduced the need for frequent irrigation in managed areas and increased water availability in natural areas; thus, larger differences in WUE between plants growing in managed vs. natural areas would be expected during normal or drier years. These results suggest that planting urban landscapes with native plants may not necessarily reduce water use because many semi‐arid plant species that are adapted to conserving water appear to have lower WUE when irrigated. These same plants also had higher leaf δ^15^N values in urban settings, presumably because periodic fertilization and irrigation increase N availability and mobility. Increases in N availability can also lead to an increase in shrub growth and/or leaf area index (Vourlitis et al. 2021), which, coupled with lower WUE, may make shrubs growing in managed areas more vulnerable to xylem embolism (Pivovaroff et al. 2016) and stem dieback (Gessler et al. 2017) if irrigation systems fail or are periodically shut off due to high water costs. In all, these data indicate that urban landscape management practices alter how plants obtain resources, which ultimately feeds back on plant physiological function and resource use. Species whose gas exchange is water or nutrient limited in natural environments might be expected to experience larger changes when exposed to management practices that alleviate these limitations. Future research should explore whether species‐specific responses are due to physiological and/or morphological plasticity and whether traits associated with water use, such as rooting depth, leaf gas exchange, and leaf morphology, can elucidate mechanisms associated with the species responses to management observed here.

## Funding

This work was supported by the Directorate for Biological Sciences (RCN:UBE 2217253).

## Conflicts of Interest

The authors declare no conflicts of interest.

## Data Availability

Data is available on the KNB database (https://knb.ecoinformatics.org/view/doi:10.5063/F11R6P1B).
